# The study of aggregation dynamics of conjugated polymer solutions in UV-vis absorbance spectra by considering the changing rate of average photon energy

**DOI:** 10.1016/j.heliyon.2021.e06638

**Published:** 2021-04-01

**Authors:** Xinyi Zhao, Peiqin Sun, Ke Zhao

**Affiliations:** a Zhengzhou University, School of Chemical Engineering, 100 Science Avenue, Zhengzhou, Henan 450002, China; b Zhengzhou University, School of Mechanics and Safety Engineering Science, 100 Science Avenue, Zhengzhou, Henan 450002, China

**Keywords:** Changing rate of average photon energy, UV-vis absorbance spectra, Aggregation process, Conjugated polymer, Poly(3-hexylthiophene), Poly(3-hexylselenophene)

## Abstract

The changing rate of average photon energy ('Eave) can describe the UV-vis absorbance spectra over a wavelength range. During the aggregation process of poly (3-hexylselenophene) (P3HS) and poly (3-hexylthiophene) (P3HT) solutions that form J-aggregates, 'Eave always decrease and the relationship between 'Eave and time is an exponential model. 'Eave can predict the time when the aggregation process is completed or how far the aggregation process is from the completion. Hansen Solubility Parameter (HSP) of the solvent can be used to predict 'Eave of some conjugated polymer solutions without doing experiments. ''E^0^ave (changing rate of 'Eave at the beginning of the aggregation process) has been calculated to reflect the overall changing trend of 'Eave and reflects the compatibility between solvent and solute. Therefore, 'Eave is suitable to describe the aggregation dynamics of conjugated polymer solutions by evaluating the aggregation process in UV-vis absorbance spectra.

## 1 Introduction

Organic optoelectronic materials are widely used these days because they have unique merits such as low cost and flexibility that traditional inorganic materials do not have [1, 2, 3, 4]. Conjugated polymer is one of the most common organic optoelectronic materials [5, 6, 7, 8, 9, 10, 11]. Normally, conjugated polymer-based devices are produced in conjugated polymer solutions and the aggregation process can influence properties of these devices [12, 13, 14, 15]. UV-vis absorption spectroscopy is one of the most commonly used method in the research of the aggregation process of conjugated polymer [16, 17, 18, 19, 20, 21, 22]. Since the aggregation process of conjugated polymer solutions is really complex with a large number of variations, it is necessary for us to study these aggregation processes systematically in-depth by studying the aggregation dynamics. Most of the papers that include the aggregation dynamics are using the amount of aggregated and soluble conjugated polymer [23, 24, 25, 26, 27, 28, 29, 30]. It can be easily known that the amount of aggregate or soluble conjugated polymer can provide us different information in a curve at a time in a UV-vis absorbance spectrum. Thus, we cannot know which one among them is the most suitable to describe the UV-vis absorbance spectra [24, 25, 26, 27, 28]. In addition, they all concentrate on only one wavelength, so they cannot reflect the situation when the maximum absorbance remains the same while the absorbance at other wavelengths change [27, 28, 29, 30]. Furthermore, the value of absorbance is related to properties of the cuvette and settings of the UV-vis spectrometer [29, 30]. Therefore, it will be necessary for researchers to find a method that can describe a UV-vis absorbance spectrum over a wavelength range.

Previously, we have obtained UV-vis absorbance spectra during the aggregation processes of poly (3-hexylselenophene) (P3HS) at different concentrations and solvents. Furthermore, we proposed two novel parameters (shape quality factor [Q] and average photon energy [Eave]) to describe the aggregation dynamics from UV-vis absorbance spectra over a wavelength range [28, 29]. Eave describes energy changes in UV-vis absorbance spectra during the aggregation process. However, these predictions are based on the regression function to obtain the predicted values at an unlimited time when the aggregation process is completed. This means that this predicted amount of aggregated conjugated polymer may not reflect the reality. In addition, we cannot know Eave change during the aggregation process accurately. Therefore, it will be helpful if we can obtain how UV-vis absorbance spectra change at any time during the aggregation process to describe the aggregation dynamics accurately.

In this paper, changing rates of average photon energy ('Eave) of our own prepared P3HS solutions and a poly (3-hexylthiophene) (P3HT) solution from another published paper are calculated to describe how the UV-vis absorbance spectra change at any time. 'Eave is obtained to predict the time when the aggregation process can be regarded as completed and how far is the aggregation process is from completion. Hansen Solubility Parameter (HSP) of the solvent is also used to predict 'Eave at the beginning of the aggregation process ('E^0^ave) so we may predict the result without doing some experiments in reality. ''E^0^ave (changing rate of 'Eave at t = 0) is calculated to describe the changing direction of 'Eave and reflect the compatibility of solvent and solute. Therefore, we will understand aggregation dynamics of conjugated polymer solutions during the aggregation process in UV-vis absorbance spectra by considering 'Eave.

### 1.1 Aggregation process of our own made P3HS solutions

i. Experimental

**Figure 1:**
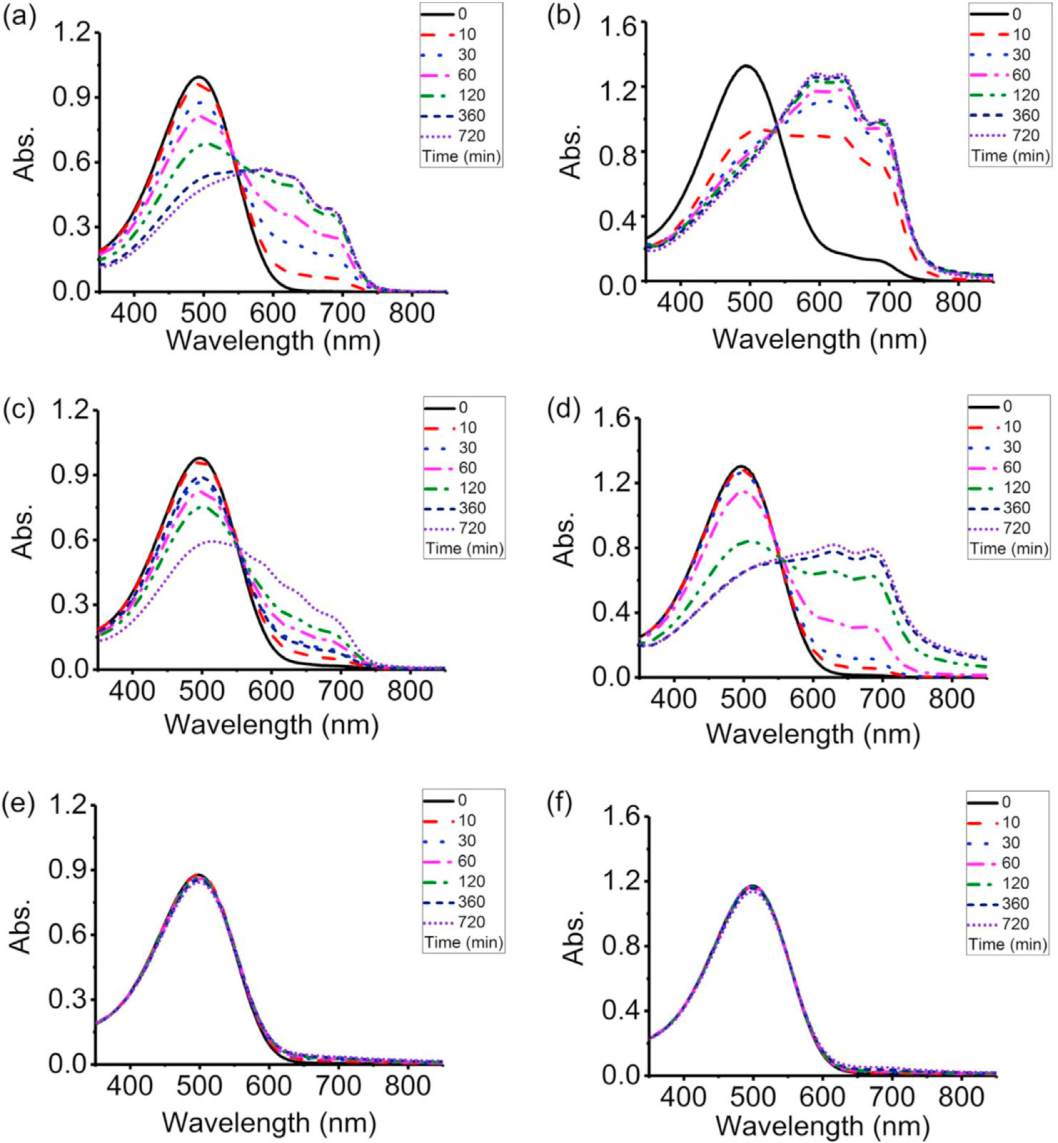
UV–vis absorbance spectra during the aggregation process of P3HS solutions: (a) 30 μg/mL toluene solution, (b) 200 μg/mL toluene solution, (c) 30 μg/mL mixed solution, (d) 200 μg/mL mixed solution, (e) 30 μg/mL chlorobenzene solution, (f) 200 μg/mL chlorobenzene solution (Cited from Figure 1 in Zhao, et al [29]).

The experimental procedures of our now made P3HS solutions can be seen in Zhao, et al [29] and they all form J-aggregates. The result is shown in Figure 1 (Cited from Figure 1 in Zhao, et al [29]).

ii. Drawbacks of traditional methods of describing UV–vis absorbance spectra.

Most of the traditional methods by describing UV–vis absorbance spectra are absorbance at a certain wavelength. They are concentrated on the absorbance at only one wavelength which definitely cannot describe the change over the whole wavelength range. In addition, absorbance at different wavelengths in the same UV-vis spectrum show the different trends during the aggregation process.

Furthermore, the most important thing that limit the use of absorbance (Abs.) is that the absorbance is related to the Beer's Law in Eq. (1):

(1) Abs. = εLC

It means that Abs. is directly proportional to ε-the molar absorptivity (a proportionality constant dependent on solvent, solute, temperature, properties of cuvette, settings of UV-vis absorbance spectrometer, etc), L-the path length (width of cuvette in this paper), C-the molar concentration. Same conjugated polymer with same concentration can have different absorbance because of different molar absorptivity and path length, so the result can hardly be repeated. As a result, the absorbance from different conditions can hardly be compared directly. In addition, many research papers even do not describe the experimental conditions in detail which make this situation even worse. Therefore, researchers definitely need a new method to solve these problems.

iii. The changing rates of average photon energy ('Eave) of P3HS solutions.

**Figure 2:**
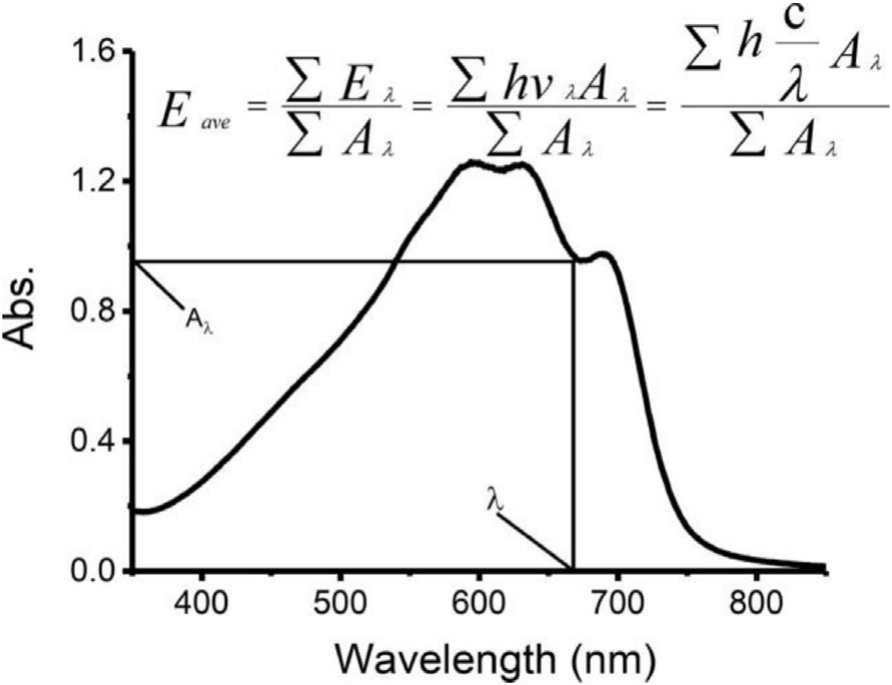
Calculation of average photon energy (Cited from Figure 4 in Zhao, et al [28]).

Details of definition, calculation and simulated model of average photon energy (Eave) for our own prepared P3HS solutions can be found in Zhao, et al [28] in Figure 2 and Eq. (2).

(2) $$\text{Eave}=\Sigma {\text{E}}_{\text{λ}}\text{/}\Sigma {\text{A}}_{\text{λ}}=\Sigma \left({\text{hv}}_{\text{λ}}{\text{A}}_{\text{λ}}\right)\text{/}\Sigma {\text{A}}_{\text{λ}}=\Sigma \left({\text{hcA}}_{\text{λ}}\text{/λ}\right)\text{/}\Sigma {\text{A}}_{\text{λ}}$$

(Cited Eq. (2) in Zhao, et al [28]).

If we consider the Beer's Law, molar absorptivity and path length do not change during the aggregation process for the same solution, it is shown in Eq. (3).

(3) Eave = ∑E_λ_/∑(εLC)_λ=_∑(hv_λ_(εLC)_λ_)/∑(εLC)_λ=_∑(hc(εLC)_λ_/λ)/∑(εLC)_λ_

Since both ∑E_λ_ and ∑A_λ_ have εLC, we can see that Eave remain the same for the same solution with different molar absorptivity and path length. Thus, Eave is independent from cuvette and UV-vis absorbance spectrometer while absorbance is not.

**Figure 3:**
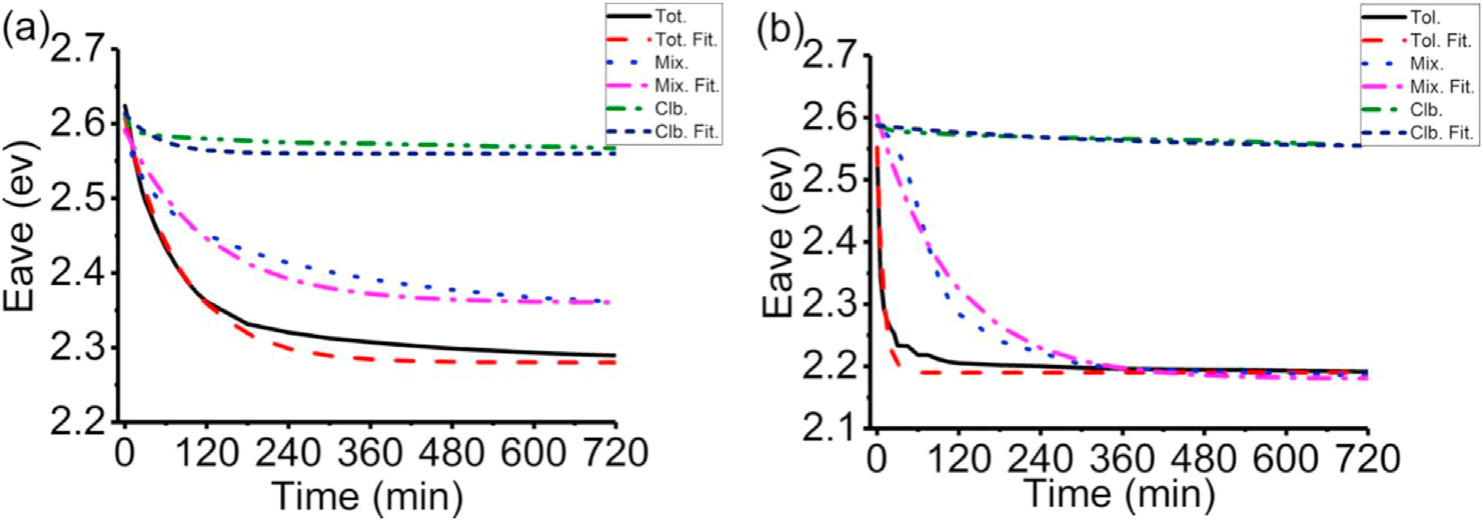
Eave for experimental UV-vis absorbance spectra during the aggregation process of P3HS solutions: (a) 30 μg/ml, (b) 200 μg/ml. Fit. means data has been fitted with a model (Cited from Figure 5 in Zhao, et al [28]).

**Figure 4:**
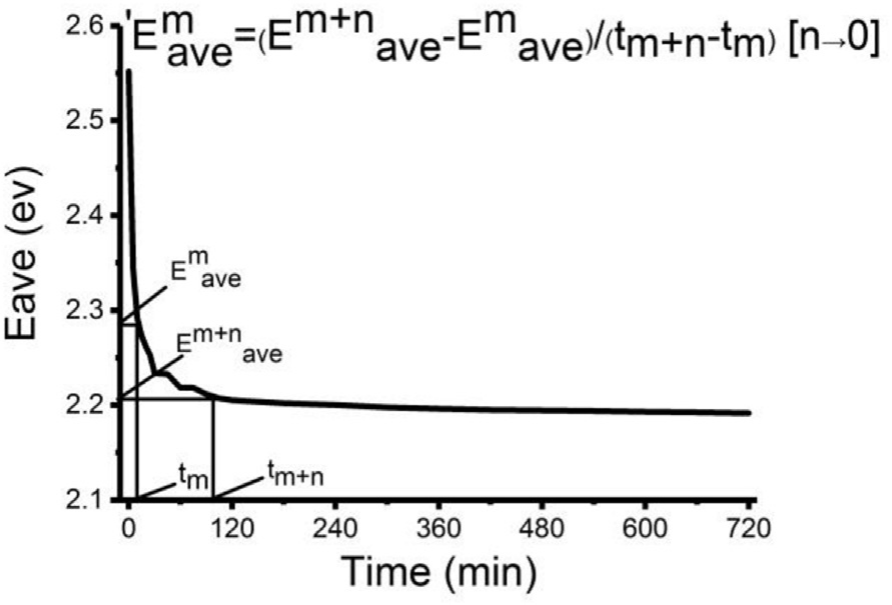
The changing rate of average photon energy ('Eave).

It can be easily known from Figure 3 that Eave does help researchers to study the aggregation process quantitatively over a wavelength range. However, it does not provide us with information how UV-vis absorbance spectra change during the aggregation process at any time accurately. We also need to study the aggregation process of P3HS solutions precisely from existing UV-vis absorbance spectra. Lastly, change of Eave when the aggregation process is completed is based on the regression model Eq. (3) E^tfit^ave = a+bexp (-ct) in Zhao, et al [28] which means it may not reflect the reality. As a result, the changing rates of Eave need to be calculated by the following function and Figure 4 and Eq. (4). It can be easily seen that 'Eave is independent from cuvette and UV-vis absorbance spectrometer.

(4) 'E^m^ave=(E^m+n^ave-E^m^ave)/(t_m+n_-t_m_) [n→0]

A mathematical model that can simulate 'Eave is obtained by us and it is shown below in Eq. (5):

(5) 'E^tfit^ave = -dexp(-ft)

'E^tfit^ave (eV/min) is the fitted changing rate of Eave at time t (min). -d (eV/min) is 'Eave at the beginning of the aggregation process (maximum 'Eave in this paper). f (min^−1^) can reflect changing rate of 'Eave. We have to know that the original 'Eave is a negative number since Eave decreases all the time during the aggregation process. However, we only display its absolute value-a positive number for comparison and avoid misunderstanding. In the paper, 'Eave always means absolute value of it.

**Figure 5:**
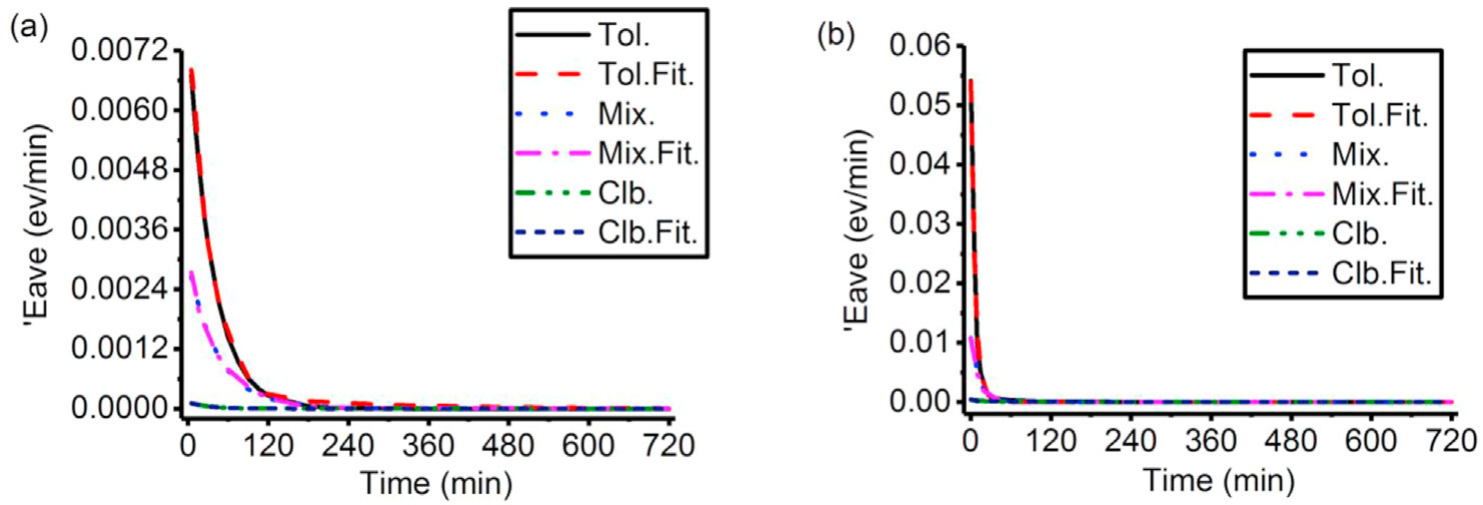
'Eave for experimental UV-vis absorbance spectra during the aggregation process of P3HS solutions: (a) 30 μg/ml, (b) 200 μg/ml.

It can be seen from Figure 5 and Table 1 that 'Eave for P3HS solutions always decrease during the aggregation process until they are around 0. 'Eave for P3HS solutions are usually higher (larger d), and decrease faster (higher f) in higher concentrations and percentage of toluene.

iv. Prediction based on the changing rates of average photon energy ('Eave) of P3HS solutions.

**Table 1:** The Fitting coefficients for the model of 'Eave of P3HS solutions.

Sol.	C.	d (eV/min)	f (min^−1^)	R.
Tol.	30	0.0067	0.0237	0.9885
	200	0.0540	0.1520	0.9959
Mix.	30	0.0025	0.0215	0.9846
	200	0.0108	0.0292	0.9904
Clb.	30	0.0002	0.0168	0.9810
	200	0.0004	0.0224	0.9872

Please note: Sol. is solvent, C. is concentration of P3HS (μg/ml), R is Pearson correlation coefficient.

It can be easily obtained from Eq. (5) that the aggregation process cannot be completed except at an infinite time. However, when 'Eave is very small compared to the maximum 'Eave during the aggregation process ('E^0^ave in this paper) (e.g. 1/10, 1/100, 1/1000, 1/10000), the change of Eave is very little that is mainly because of random variations. Thus, these aggregation processes may be regarded as completed at a limited time according to our practical needs. In addition, we will know how far a UV-vis absorbance spectrum during the aggregation process is from completion. Therefore, we calculate time when 'Eave reaches a really small proportion of 'Eave at the beginning of the aggregation process in Table 2. It shows the whole process of how fully soluble solutions have been transformed into solutions with aggregates.

**Table 2:** Time when 'E^fit^ave reach a small percentage of 'E^0^ave with a model of P3HS solutions.

Sol.	C.		'E^0^ave	1/10 'E^0^ave	1/100 'E^0^ave	1/1000 'E^0^ave	1/10000 'E^0^ave	Infinite time
Tol.	30	Time (min)	0	97	194	291	389	Infinite
		'E^fit^ave (eV/min)	6.73E-3	6.73E-4	6.73E-5	6.73E-6	6.73E-7	0
		E^fit^ave (eV)	2.6110	2.3111	2.2813	2.2783	2.2780	2.2780
	200	Time (min)	0	15	30	45	61	Infinite
		'E^fit^ave (eV/min)	5.43E-2	5.43E-3	5.43E-4	5.43E-5	5.43E-6	0
		E^fit^ave (eV)	2.5240	2.2071	2.1755	2.1724	2.1720	2.1720
Mix.	30	Time (min)	0	107	214	321	428	Infinite
		'E^fit^ave (eV/min)	2.72E-3	2.72E-4	2.72E-5	2.72E-6	2.72E-7	0
		E^fit^ave (eV)	2.5820	2.3741	2.3533	2.3512	2.3510	2.3510
	200	Time (min)	0	79	158	237	315	Infinite
		'E^fit^ave (eV/min)	1.08E-2	1.08E-3	1.08E-4	1.08E-5	1.08E-6	0
		E^fit^ave (eV)	2.6440	2.2300	2.1886	2.1845	2.1840	2.1840
Clb.	30	Time (min)	0	137	274	411	548	Infinite
		'E^fit^ave (eV/min)	1.88E-4	1.88E-5	1.88E-6	1.88E-7	1.88E-8	0
		E^fit^ave (eV)	2.5900	2.5648	2.5623	2.5620	2.5620	2.5620
	200	Time (min)	0	103	206	308	411	Infinite
		'E^fit^ave (eV/min)	3.80E-4	3.80E-5	3.80E-6	3.80E-7	3.80E-8	0
		E^fit^ave (eV)	2.5850	2.5535	2.5504	2.5500	2.5500	2.5500

Table 2 shows that 'E^fit^ave will always decrease to 1/10000 of 'E^0^ave before 720 min and Eave does not change after 'E^fit^ave is below 1/10000 of 'E^0^ave. Therefore, there will be no need for us to continue the experiment of aggregation process and the aggregation process can be regarded as completed at this time for our need. At this time, 'E^fit^ave is nearly 0 and E^fit^ave is really near E^fit^ave at the unlimited time.

We can see that 'Eave follow the similar model-exponential function with different coefficients. We can find that Eave and 'Eave can reflect the whole curve at a time in a UV-vis absorbance spectrum. In addition, a curve at the corresponding time in a UV-vis absorbance spectrum only has one Eave and 'Eave.

v. Relationship between Hansen Solubility Parameter (HSP) and 'Eave.

From the above paragraphs we can see that 'Eave for P3HS solutions with different solvents and concentrations can differ much. However, there is no quantitative reason to describe and predict 'Eave. HSP is a well-known and suitable parameter to describe compatibility between solvent and solute-which is important for solubility. Thus, HSP can also influence the aggregation rate much [31, 32]. We have already known how to calculate HSP for each solvent and polymer. As a result, it will be easy for us to relate aggregation rate with the difference of HSP between a solvent and P3HS.

According to the calculation method in Chen, et al [33], the HSP of P3HS is 19.79. Please note that all HSP in our paper are δ_T_, where δ_T_^2^ = δ_D_^2^+δ_P_^2^+δ_H_^2^. HSP of toluene and chlorobenzene are 18.16 and 19.58 according to the Hansen, [34]. HSP of mix solvent with the volume ratio of toluene and chlorobenzene = 1:1 is 18.82. E^0^ave can be obtained from the experimental data directly and it is independent from regression models. As a result, we can find a relationship between HSP and 'Eave at the beginning of the aggregation process in Eq. (6).

(6) 'E^0hfit^ave = -gj^k^

'E^0hfit^ave (eV/min) is 'Eave at the beginning of the aggregation process (maximum 'Eave in this paper). j is the absolute value of the difference between HSP of P3HS and solvent (For example, if HSP of P3HS and toluene are 19.79 and 18.16, j = |19.79-18.16| = 1.63). g (eV/min) is a coefficient. k is a coefficient which reflects sensitivity of 'E^0hfit^ave to the change of j. g, k can be modified to increase the Pearson correlation coefficient.

**Figure 6:**
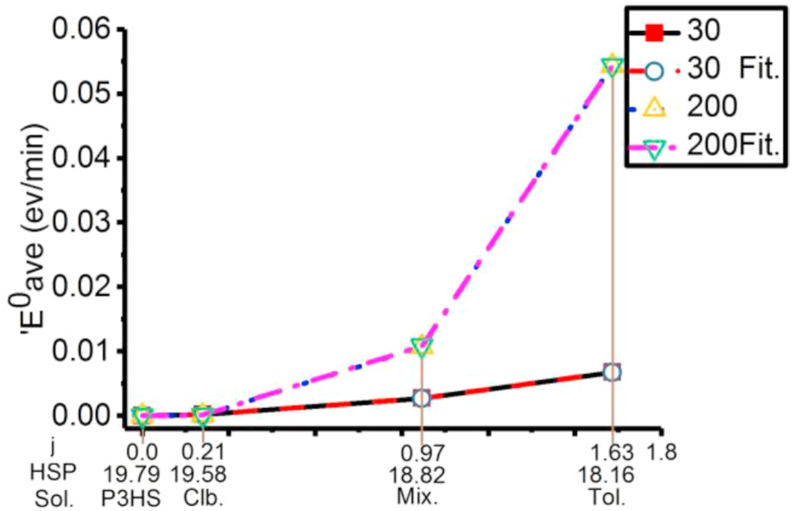
'E^0hfit^ave and 'E^0^ave for UV-vis absorbance spectra during the aggregation process of P3HS solutions.

For 30 μg/ml P3HS solutions: g = 0.0029 eV/min k = 1.755, R = 0.9965. for 200 μg/ml: g = 0.0119 eV/min, k = 3.106, R = 0.9953. It can be easily seen from Figure 6 and Pearson correlation coefficients that 'E^0hfit^ave really fits 'E^0^ave well for P3HS solutions at different solvents and concentrations. It shows that we can predict 'E^0^ave without doing experiment for some solvents with the same concentration of P3HS. This also helps us to make plan for our experiments before we actually start. There is no doubt that this will save much time, money, resources and labor. Please note that one conjugated polymer such as P3HS can have different structures can be different so we can hardly use experimental data from different papers directly.

Since we just have done experiments on two concentrations, we lack data to obtain rules of relating concentration with 'Eave. Furthermore, UV-vis absorbance spectra of solutions of different concentrations are usually performed at different conditions because of the Beer's Law and absorbance range of the UV-vis absorbance spectrometer. In addition, it will be easier to perform UV-vis absorbance spectra of conjugated polymer with the different concentrations than different solvents. As a result, it will be nearly impossible and little value to quantitatively relate concentration with parameters of 'Eave.

Therefore, we can know aggregation dynamics of conjugated polymer solutions during the aggregation process in UV-vis absorbance spectra by 'Eave and the details are as follows:

1) Calculate Eave of the UV-vis absorbance spectra of a conjugated polymer solution during the aggregation process.
2) Calculate 'Eave based on Eave obtained from the UV-vis absorbance spectra.
3) Use simulated formula to get when 'Eave will reach a tiny ratio of maximum 'Eave.
4) Calculate corresponding Eave of 'Eave in 3) to see how far it is from completion.
5) Use Eq. (6) to predict 'E^0^ave of other solvents at the same concentration of conjugated polymer.
'Eave can be used in aggregation processes of conjugated polymer solutions under a variety of conditions.

### 1.2 Aggregation process of a P3HT solution in a published paper

As stated previously, we can conclude that 'Eave is excellent to describe the aggregation process of P3HS solutions in UV-vis absorbance spectra. However, it does not prove that 'Eave also suits other conjugated polymer solutions during the aggregation process well. To solve this question, we choose to calculate a well-known conjugated polymer-P3HT solution (5 μg/ml P3HT mixed solution of volume ratio of toluene: acetonitrile = 90:10 [35]).

We choose to calculate a 5 μg/ml P3HT mixed solution of volume ratio of toluene: acetonitrile = 90:10 (Figure 8(a) in Johnson, et al [35]), because curves of different times in that Figure could be easily distinguished and this P3HT solution also forms J-aggregates. It records UV-vis absorbance spectra at aggregation time from 0-75 min at the time interval of 5 min and UV-vis absorbance spectra at 24 h.

i. Average photon energy (Eave) and its changing rate in a P3HT solution of a 5 μg/ml P3HT mixed solution of volume ratio of toluene: acetonitrile = 90:10.

**Figure 7:**
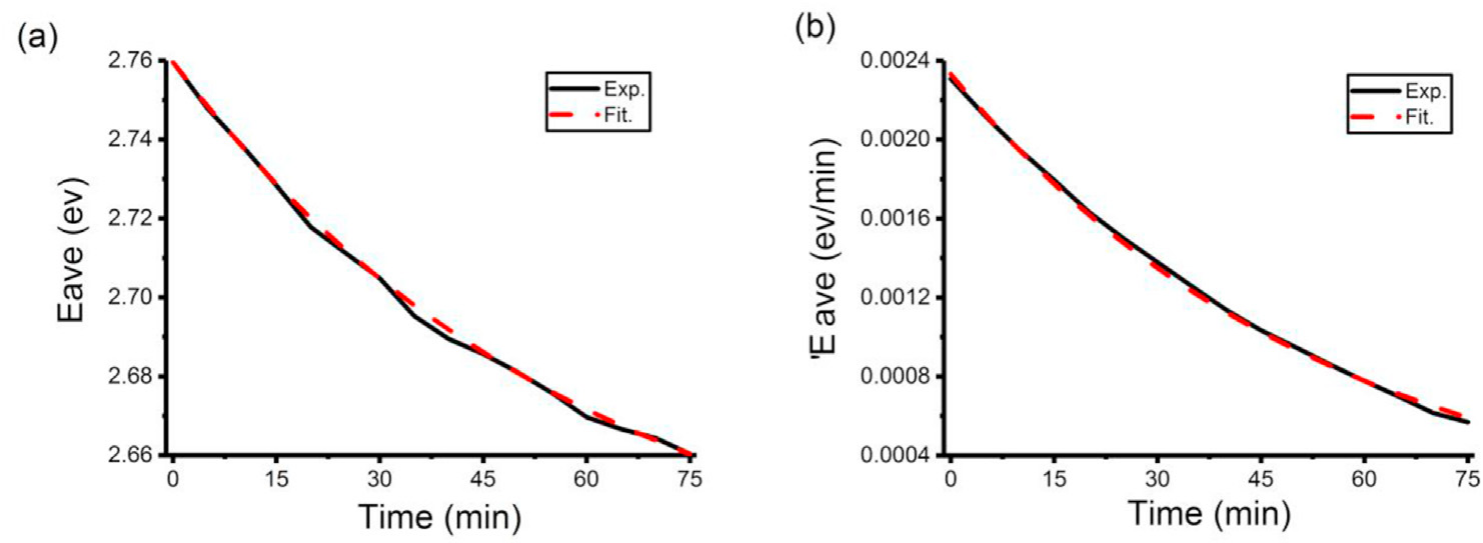
The Eave and 'Eave of a 5 μg/ml P3HT mixed solution of volume ratio of toluene: acetonitrile = 90:10 during the early stage (0–75 min) of aggregation process [(a) Eave, (b) 'Eave]. Exp. means data are obtained from experiment directly while Fit. means data have been fitted by a model.

It could be easily found in Figure 7 (a) that Eave for this P3HT solution follow nearly the same rules to them for our own prepared P3HS solutions-an exponential model: Eq. (3) E^tfit^ave = a+bexp (-ct) in Zhao, et al [28]. Coefficients in this model are a = 2.6211 eV, b = 0.1386 eV, c = 0.0168 min^−1^. This model has a really high Pearson correlation coefficient (0.9962). What can be known from Figure 7 (b) is that 'Eave for this P3HT solution follow similar rules to them for our own prepared P3HS solutions-an exponential model: Eq. (5) 'E^tfit^ave = -dexp (-ft). In this model, d = 0.0023 eV/min, f = 0.0183 min^−1^. It also has a very high Pearson correlation coefficient of 0.9989.

ii. Prediction based on the changing rates of average photon energy ('Eave) of a 5 μg/ml P3HT mixed solution of volume ratio of toluene: acetonitrile = 90:10.

From Table 3 we can see that 'E^fit^ave will drop to only 1/10000 of 'E^0^ave at only 504 min and E^fit^ave does not change after that. However, at 75 min 'E^fit^ave is larger than 1/10 of 'E^0^ave. This means that it is not suitable for us to stop observing the aggregation process at 75 min, but there is no need for us to continue the aggregation process to 24 h.

**Table 3:** Time when 'E^fit^ave reach a small percentage of 'E^0^ave with a model of a 5 μg/ml P3HT mixed solution of volume ratio of toluene: acetonitrile = 90:10.

	'E^0^ave	1/10 'E^0^ave	1/100 'E^0^ave	1/1000 'E^0^ave	1/10000 'E^0^ave	end of experiment: 24 h
Time (min)	0	126	252	378	504	1440
'E^fit^ave (eV/min)	2.3E-2	2.3E-3	2.3E-4	2.3E-5	2.3E-6	8.7E-15
E^fit^ave (eV)	2.7597	2.6350	2.6225	2.6213	2.6211	2.6211

From these P3HS and P3HT solutions that form J-aggregates, we can see that 'Eave is a suitable to describe the change of UV-vis absorbance spectra over a wavelength range at any time during the aggregation process. Thus, studying aggregation dynamics of conjugated polymer solutions during the aggregation process in UV-vis absorbance spectra by 'Eave is excellent for aggregation processes of different conjugated polymer solutions under many conditions.

### 1.3 The changing rate of 'Eave (''Eave) at the beginning of the aggregation process

Now we have discussed a lot about 'Eave of conjugated polymer solutions during the aggregation process. However, since 'Eave always changes during the aggregation process so its changing rate is not 0 and also changes during the aggregation process as the following equation: Eq. (7):

(7) ''E^tfit^ave = d'Eave/dt

Because Eq. (5) 'E^tfit^ave = -dexp (-ft) and its Pearson correlation coefficient is always high (>0.99), ''Eave can be fitted by Eq. (8):

(8) ''E^tfit^ave = dfexp(-ft)

At the beginning of the aggregation process, ''E^0fit^ave = df and it can provide people with the overall value of ''Eave during the aggregation.

**Figure 8:**
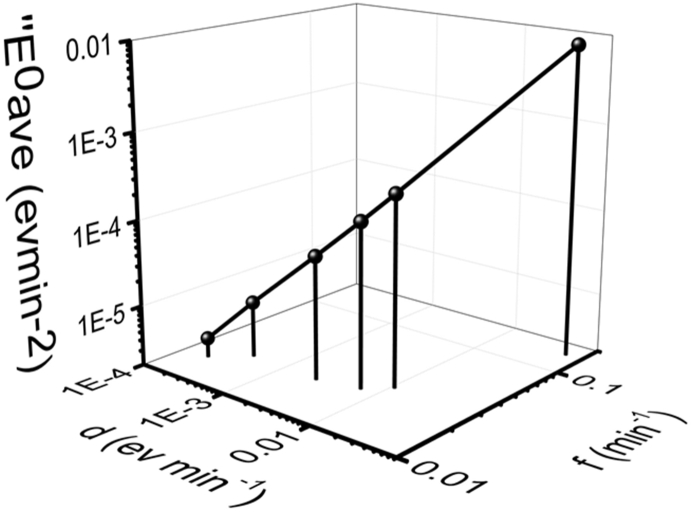
Relationship between ''^0^Eave and 'Eave of P3HS solutions.

**Figure 9:**
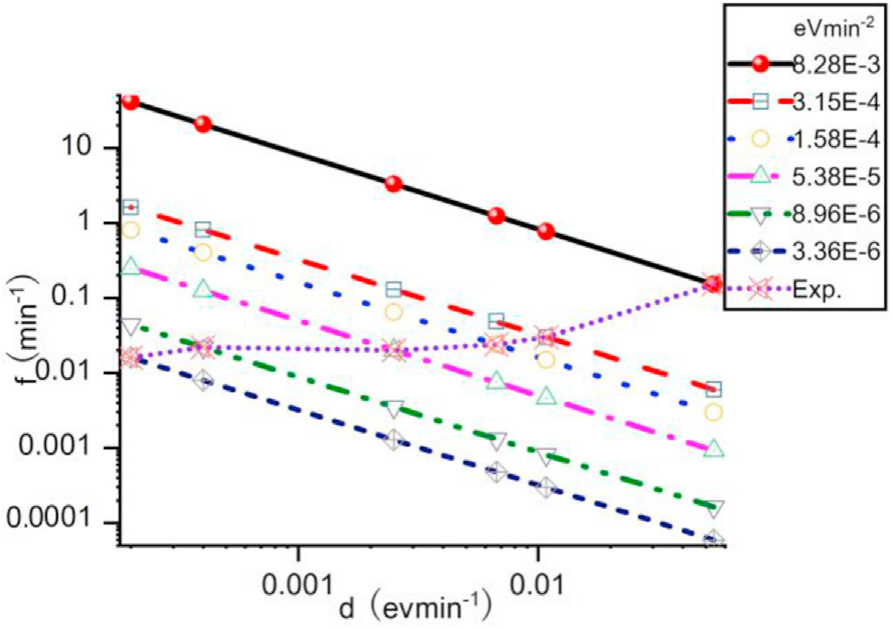
The same ''E^0^ave with different combinations of d and f. The line (Exp.) means points in this are obtained directly from experiments while other lines mean the same ''E^0^ave.

Because the ranges of d and f are too large (max/min>1000), Figure 8 and Figure 9 are drawn in logarithm scale. Thus, points in Figure 9 represent the same ''E^0^ave are in a straight line. It can be easily seen from Table 4 that higher d usually means higher f (except for 30 μg/ml mixed solution, d [30 μg/ml mixed]>d [200 μg/ml chlorobenzen], but f [30 μg/ml mixed]<f [200 μg/ml chlorobenzen]). ''E^0^ave always increases when d increase. Conjugated polymer solution with higher ''E^0^ave (longer distance between the line with same ''E^0^ave and the origin point (d = 0, f = 0) usually means worse compatibility between the solvent and the solute. For 5 μg/ml P3HT mixed solution (Figure 8(a) in Johnson, et al [35]), d = 0.0023 eVmin^-1^, f = 0.0183 min^−1^, ''E^0^ave = 4.21E-5 eVmin^-2^.

**Table 4:** Relationship between ''E^0^ave and 'Eave of P3HS solutions.

Sol.	C.	d (eVmin^−1^)	f (min^−1^)	''E^0^ave (eVmin^−2^)
Tol.	30	0.0067	0.0237	1.58E-4
	200	0.0540	0.1520	8.28E-3
Mix.	30	0.0025	0.0215	5.38E-5
	200	0.0108	0.0292	3.15E-4
Clb.	30	0.0002	0.0168	3.36E-6
	200	0.0004	0.0224	8.96E-6

As a result, ''E^0^ave is a suitable parameter to describe the aggregation process and is suitable to reflect the overall changing trend of 'Eave.

## 2 Discussion

In this paper, we have used Hansen Solubility Parameter (HSP) to predict 'E^0^ave with different solvents while other conditions remain the same. However, in Eq. (5) 'E^tfit^ave = -dexp (-ft), d = 'E^0^ave. f is for increasing Pearson correlation coefficient and it is obtained by the regression exponential model. HSP is a thermodynamic parameter that is independent from concentration and time. It will be difficult to correlate HSP with f in Eq. (5) 'E^tfit^ave = -dexp (-ft),. However, 'Eave is mainly determined by the solute (conjugated polymer), solvent, concentration, temperature. If other conditions are unchanged, we will be able to correlate solvent (includes its HSP and other properties) with f in Eq. (5) 'E^tfit^ave = -dexp (-ft). However, there is no doubt that more experiments and analysis must be performed. In addition, UV-vis absorbance spectra from other related research papers should be collected and compared to get a conclusion that fits the reality well.

For other conditions that can influence 'Eave much-concentration of conjugated polymer, more works must be done. An UV-vis absorbance spectrometer with large sensitive absorbance range is needed and UV-vis absorbance spectra for a conjugated polymer with different concentrations must be done with other conditions remain the same. Many experiments with analysis should be completed and UV-vis absorbance spectra from other related resources must be also used before researchers can get a result that reflects the reality. Other conditions such as temperature, conjugated polymer type follow the similar procedure.

Previously, ''E^0^ave has been calculated to reflect the overall changing trend of 'Eave. However, if we would like to study the change of 'Eave at any time during the aggregation process in-depth, time-dependent ''Eave must be obtained. Researchers have to compare ''Eave directly got from experimental data and fitted Eave. There is no doubt that more experiments and analysis of more conditions (temperature, concentration, conjugated polymer, solvent, etc) should be done before people can get a reliable conclusion.

## 3 Conclusion

The changing rate of average photon energy ('Eave) is an excellent parameter to describe the aggregation dynamics of conjugated polymer solutions in UV-vis absorbance spectra. It has advantages (unique and over a wavelength range) over traditional ones that just concentrate on absorbance at one wavelength. It can predict the time when the aggregation process is completed or how far a UV-vis absorbance spectrum during the aggregation process is from completion. Changing rates of average photon energy ('Eave) are calculated to UV-vis absorbance change at any time during the aggregation process. 'Eave always decreases during the aggregation process of P3HS solutions. The relationship between 'Eave and time is always an exponential model. Hansen Solubility Parameter (HSP) of the solvent can be used to predict 'Eave of P3HS solutions at different solvents at the same concentration. P3HT solutions of different conditions follow the same rules of P3HS solutions during the aggregation process. ''E^0^ave (changing rate of 'Eave at the beginning of the aggregation process) is obtained to describe the overall changing trend of 'Eave and compatibility between solvent and solute. In conclusion, 'Eave is suitable for the study of aggregation dynamics of conjugated polymer solutions that form J-aggregates in UV-vis absorbance spectra.

## Declarations

### Author contribution statement

Xinyi ZHAO: Conceived and designed the experiments; Performed the experiments; Analyzed and interpreted the data; Wrote the paper.

Peiqin SUN: Analyzed and interpreted the data; Contributed reagents, materials, analysis tools or data.

Ke ZHAO: Contributed reagents, materials, analysis tools or data; Wrote the paper.

### Funding statement

This research did not receive any specific grant from funding agencies in the public, commercial, or not-for-profit sectors.

### Data availability statement

The authors are unable or have chosen not to specify which data has been used.

### Declaration of interests statement

The authors declare no conflict of interest.

### Additional information

No additional information is available for this paper.
